# New recommendations for the composition of influenza vaccines in 2023 for the southern hemisphere

**Published:** 2022-10

**23 September 2022** - Geneva - The World Health Organization has announced the recommended viral composition of influenza vaccines for the 2023 southern hemisphere influenza season.

The recommendations issued today will be used by national vaccine regulatory agencies and pharmaceutical companies to develop, produce, and license influenza vaccines for the following influenza season. The periodic update of viruses contained in influenza vaccines is necessary for the vaccines to be effective due to the constantly evolving nature of influenza viruses.

The recommendation is based on the advice of a group of experts from WHO Collaborating Centres and WHO Essential Regulatory Laboratories that analyze virus surveillance data generated by the WHO Global Influenza Surveillance and Response System (or GISRS).

Around a billion people get seasonal influenza every year and the threat of an influenza pandemic is ever-present. For this reason, the need to monitor circulating respiratory viruses, including influenza, continues to be critical. This monitoring informs the vaccine composition recommendations that WHO issues twice a year.

The year-round surveillance is conducted by GISRS, a global network of over 150 laboratories in 127 countries, areas or territories set up in 1952. This year we celebrate its 70th anniversary.

Going forward, GISRS will continue to use its unique position as a global respiratory surveillance network to add value to other respiratory virus threats, including COVID-19, where it has already played a significant role. It will also make use of emerging technologies, for example by expanding genomic surveillance, to continue to protect people from the threat of influenza.

**Recommendations**

WHO recommends that quadrivalent vaccines for use in the 2023 southern hemisphere influenza season contain the following:

**Egg-based vaccines**

- an A/Sydney/5/2021 (H1N1)pdm09-like virus;
- an A/Darwin/9/2021 (H3N2)-like virus;
- a B/Austria/1359417/2021 (B/Victoria lineage)-like virus; and
- a B/Phuket/3073/2013 (B/Yamagata lineage)-like virus.

**Cell culture- or recombinant-based vaccines**

- an A/Sydney/5/2021 (H1N1)pdm09-like virus;
- an A/Darwin/6/2021 (H3N2)-like virus;
- a B/Austria/1359417/2021 (B/Victoria lineage)-like virus; and
- a B/Phuket/3073/2013 (B/Yamagata lineage)-like virus.

WHO recommends that trivalent vaccines for use in the 2023 southern hemisphere influenza season contain the following:

**Egg-based vaccines**

- an A/Sydney/5/2021 (H1N1)pdm09-like virus;
- an A/Darwin/9/2021 (H3N2)-like virus; and
- a B/Austria/1359417/2021 (B/Victoria lineage)-like virus.

**Cell culture- or recombinant-based vaccines**

- an A/Sydney/5/2021 (H1N1)pdm09-like virus;
- an A/Darwin/6/2021 (H3N2)-like virus; and
- a B/Austria/1359417/2021 (B/Victoria lineage)-like virus

Available from: https://www.who.int/news/item/23-09-2022-new-recommendations-for-the-composition-of-influenza-vaccines-in-2023-for-the-southern-hemisphere

